# The social and ecological costs of an ‘over-extended' phenotype

**DOI:** 10.1098/rspb.2015.2359

**Published:** 2016-01-13

**Authors:** Lyndon Alexander Jordan, Sean M. Maguire, Hans A. Hofmann, Masanori Kohda

**Affiliations:** 1 Department of Collective Behaviour Max Planck Institute for Ornithology, University of Konstanz, Konstanz, Germany; 2 Laboratory of Animal Sociology, Department of Biology and Geosciences, Graduate School of Science, Osaka City University, Japan; 3 Department of Integrative Biology, The University of Texas at Austin, Austin, TX, USA; 4 Institute for Cellular and Molecular Biology, Institute for Neuroscience, The University of Texas at Austin, Austin, TX, USA

**Keywords:** attractiveness, territory, cichlid, mate choice, predation, extended phenotype

## Abstract

Extended phenotypes offer a unique opportunity to experimentally manipulate and identify sources of selection acting on traits under natural conditions. The social cichlid fish *Neolamprologus multifasciatus* builds nests by digging up aquatic snail shells, creating an extended sexual phenotype that is highly amenable to experimental manipulation through addition of extra shells. Here, we find sources of both positive sexual selection and opposing natural selection acting on this trait; augmenting shell nests increases access to mates, but also increases social aggression and predation risk. Increasing the attractiveness of one male also changed social interactions throughout the social network and altered the entire community structure. Manipulated males produced and received more displays from neighbouring females, who also joined augmented male territories at higher rates than unmanipulated groups. However, males in more attractive territories received more aggression from neighbouring males, potentially as a form of social policing. We also detected a significant ecological cost of the ‘over-extended' phenotype; heterospecific predators usurped augmented nests at higher rates, using them as breeding sites and displacing residents. Using these natural experiments, we find that both social and ecological interactions generate clear sources of selection mediating the expression of an extended phenotype in the wild.

## 1. Introduction

A fundamental tenet of biology posits that the benefits of sexual traits will be balanced by the costs of producing or maintaining them [1]. A vast body of work is dedicated to measuring these costs and benefits, yet experimentally testing how these may vary with changing trait values is challenging for a number of reasons. First, benefits of sexual traits typically arise in the form of increased access to mates [2,3], which can be difficult to measure when, as is the case in many species, pairing between mates is brief and requires continual observation to accurately measure. Second, the costs associated with increased trait values can be difficult to measure in the wild, chronic costs may be paid incrementally over a longer period than can be experimentally captured [4]. Moreover, catastrophic costs such as mortality associated with predation can be a primary source of selection acting on sexual traits [5], but by definition occur once in the lifetime of an individual and so may be difficult to directly observe. To quantify changes associated with increased trait values requires both very accurate tracking of individuals (in itself a challenge under natural conditions) and large sample sizes of manipulated individuals, but experimental manipulations of traits in their natural environment can generate valuable insights into the evolution of sexual traits generally.

Model systems where the sexual trait of interest is part of an extended phenotype are particularly promising in this regard. Extended phenotypes provide an extremely useful tool to examine the ecological costs of increasing attractiveness as they can be manipulated independently of other traits, thus minimizing uncontrolled effects on other aspects of the same or related traits [6–8]. Of even greater value is the potential to experimentally ‘over-extend’ phenotypes beyond that which is possible for bodily traits. This could reveal sources of selection that are either (i) obscured by more proximate factors such as resource allocation and physiological trade-offs, or (ii) specific only to extended phenotypes and therefore vital to our understanding of the evolution of these traits. Despite their obvious utility for studying the evolution of sexual traits, the use of extended phenotypes remains limited to a handful of species [6]. The best-studied examples of extended phenotypes as sexual signals are found in bowerbirds, in which bower ornamentation predicts mating success [9]. However, this increase does not always yield a net benefit, as social control of signal expression occasionally results in conspecific destruction of bowers [10,11]. Indeed, social feedback and competition with conspecifics appear to represent an important limitation on the unbounded increase of extended sexual phenotypes. Social punishment of ‘cheaters' is found in many social systems [12] and has also been observed in the context of sexual signals. Males possessing sexual traits that do not match with other physical indicators or which cannot be defended are subject to increased aggression from social group members [13,14]. Finally, larger nests or territories in many taxa can increase exposure to predation [15,16], thereby potentially limiting their size in natural conditions [17].

To understand how the sources of selection on extended phenotypes may differ in strength and form from those commonly reported for bodily traits, we conducted experiments with the highly social cichlid fish *Neolamprologus multifasciatus* in Lake Tanganyika, manipulating a sexual trait that is part of the animal's extended phenotype. In this species, males build nests by uncovering empty snail shells from the sediment that the much smaller females use as breeding sites. As is the case for numerous bird species, the nests serve multiple ecological functions [18], providing shelter and a nesting site as well as attracting mates (for a detailed natural history of the study species, see the electronic supplementary material). Prior work has shown that experimental addition of shells to male nests increases attractiveness [19,20] and sexual selection therefore favours males with more shells. However, field observations reveal that nest size of wild males is consistently smaller than males in captivity [21]. This is despite the fact that empty shells are an apparently unlimited resource under natural conditions (see electronic supplementary material, movie S1), and males appear to be able to extend their territories. To examine the seemingly paradoxical limitation males place on their own attractiveness, we studied the social and ecological consequences of *in situ* manipulations of territory size and quality. In particular, we assessed (i) whether increasing the attractiveness of a male's territory causes him to attract more females, indicating positive sexual selection acting this trait; (ii) if this in turn causes extra subordinate males to join the group, potentially diluting the benefits of attracting new females; (iii) whether males with experimentally more attractive nests are subject to increased social aggression; and (iv) whether males with experimentally more attractive nests are subject to higher ecological risk in the form or territory loss or predation.

## 2. Material and methods

This study was conducted in Lake Tanganyika near Mpulungu, Zambia (8.7667° S, 31.1333° E), between 2 October 2011 and 30 November 2011. *Neolamprologus multifasciatus* is a Lake Tanganyikan cichlid fish species that lives in stable social groups on beds of empty *Neothauma* gastropod shells [21], which are used both for shelter from predators and as brood chambers in which the smaller females deposit eggs [22]. Social groups are composed of one to three adult males, up to five adult females and offspring from different clutches [20]. Neighbouring social groups are typically less than 30 cm apart and can be part of large colonies composed of hundreds of social units. A representative community and habitat are shown in electronic supplementary material, movie E1, and a detailed natural history can be found in electronic supplementary material, S1. Individuals can be identified by the pattern of vertical bars on their flanks, which is further aided by the site fidelity of these fish as they only rarely moved more than 30 cm from their territory. In our experiments, when immediate identification was ambiguous, we referred back to video recordings taken during observations (Sony G12 camera) to later confirm the identity of individuals. Data were collected with direct observations while SCUBA diving at depths between 8 and 11 m over an 8-day period for each group.

### (a) Social network analysis

Networks were created for each community on each day separately using the R package, igraph [23]. Nodes represent individuals, while edge weights represent the number of interactions among individuals. We constructed separate networks for four different interactions types: in-degree from males, in-degree from females, out-degree towards males, out-degree towards females. These four metrics broadly defined aggression among males and affiliative behaviour among males and females, and were aggregates of behaviours detailed in electronic supplementary material, S5. Our network analyses followed recommendations outlined by James *et al.* [24]. We analysed whole network autocorrelation patterns across time, correlating all consecutive days of each network to calculate product moment correlation coefficients of the network adjacency matrices. We then assessed the significance of these coefficients using a modified form of the quadratic assignment procedure (for details see electronic supplementary material, S2) implemented in the SNA R package (1000 replicates; [25]). To provide a more realistic and conservative null distribution, we first identified community structure in the networks and then restricted the label swapping of the nodes to within sub-group as defined by the Walktrap algorithm (see electronic supplementary material, S2; [26]). *p*-values are given as the proportion of those randomized replicates that have a greater correlation coefficient than the observed correlation coefficient, with the null hypothesis that the two matrices are unrelated. In- and out-degree densities were compared separately for intra- and inter-sexual interactions with a two factor GLMM with a Poisson error distribution that included day and treatment as fixed effects, with fish identity nested within communities as random effects. In addition, we included an observation-level random effect to model over-dispersion in our count data (also known as a Poisson-lognormal model; [27]). In electronic supplementary material, S3, we tested for effects of distance from the manipulated territory and territory size on networks metrics.

### (b) Part II: increased access to mates and ecological costs of increased territory attractiveness

Experimental groups were chosen to contain a single male and either one or two adult females (one female *N* = 78, two females *N* = 95), and one, two or no juveniles (no juveniles *N* = 51, one juvenile *N* = 85, two juveniles *N* = 37). The mean number of males, females and juveniles per group was not significantly different among treatments (ANOVA, females *F*_d.f._ = 0.562_6,166_, *p* = 0.760; juveniles *F*_d.f._ = 0.243_6,166_, *p* = 0.851).

Prior to manipulation, experimental groups were selected and identified by placing small pieces of flat stone marked with treatment number adjacent to the nest depression. Experimental groups were at least 1 m apart and were never nearest neighbours with another experimental group. The location of groups was tracked by recording digital video on line transects (2 m × 10–15 m) throughout the experimental area and later drawing a scale map. For each group, we recorded the number of males, females and juveniles (small individuals without vertical bars) and the number of visible (i.e. uncovered and above the sand line) broken or intact shells. To confirm that females were the smaller barred individuals within each group, a subset of individuals (*n* = 30) was collected after termination of trials and vent-sexed. In all cases, visual sex identification was confirmed by vent-sexing.

Experimental manipulations consisted of six treatment groups and three control groups. Treatments R2 and R4 consisted of replacing two or four broken shells in the group with intact shells. Treatments A2 and A4 consisted of adding two or four intact shells to each group. Treatments AR2 and AR4 consisted of both replacing and adding two or four shells to each group. The territory area was not increased with any manipulations, which were all made within the nest depression. In control treatment C1, groups were left completely untouched, in treatment C2 four broken shells were added to each group, and in treatment C3 four broken shells were replaced with four different broken shells. Each group was observed five times. The initial observation occurred before experimental manipulation to record baseline demographic data within groups. The subsequent four observations took place at 3-day intervals, such that the total time between the initial and final observations was 12 days. At each observation, the number of males, females and juveniles within each group was recorded, as well as the presence of heterospecifics inside the territory.

### (c) Statistical analysis

To investigate the overall effect of treatment on the frequency of territory takeover by heterospecifics, we used Bayesian logistic regression with treatment as the only factor and territory loss (1 = territory lost, 0 = territory kept) as the response [28]. Post hoc tests were done on nine planned contrasts using least-squares means. Each treatment was compared to the corresponding controls, such that replacing treatments (R2 and R4) were compared to replacement control C2, adding treatments (A2 and A4) were compared to adding control C3 and combination treatments (AR2 and AR4) were compared against pooled C2 and C3 control groups. Additionally, we compared R2 against R4, A2 against A4, and AR2 against AR4 to test the effects of adding additional shells. In an alternate analysis (electronic supplementary material, S4), we compared all treatments against the pooled controls and found concordant results.

Changes in the number of males and females were performed on the subset of communities that were not lost by observers or taken over by heterospecifics during the observation period (*N*: C1 = 18, C2 = 18, C3 = 18, R2 = 18, R4 = 18, A2 = 20, A4 = 10, AR2 = 15, AR4 = 12). We calculated the change by subtracting the number of individuals present in the last observation from the number observed in the first observation. We used Bayesian linear models with treatment as a fixed factor and post hoc testing where appropriate on the same nine planned contrasts described above. All *p*-values have been corrected for multiple comparisons using false discovery rate (FDR) corrections [28].

## 3. Results

### (a) Part I: social network analysis of increased territory attractiveness

**Figure 1.**
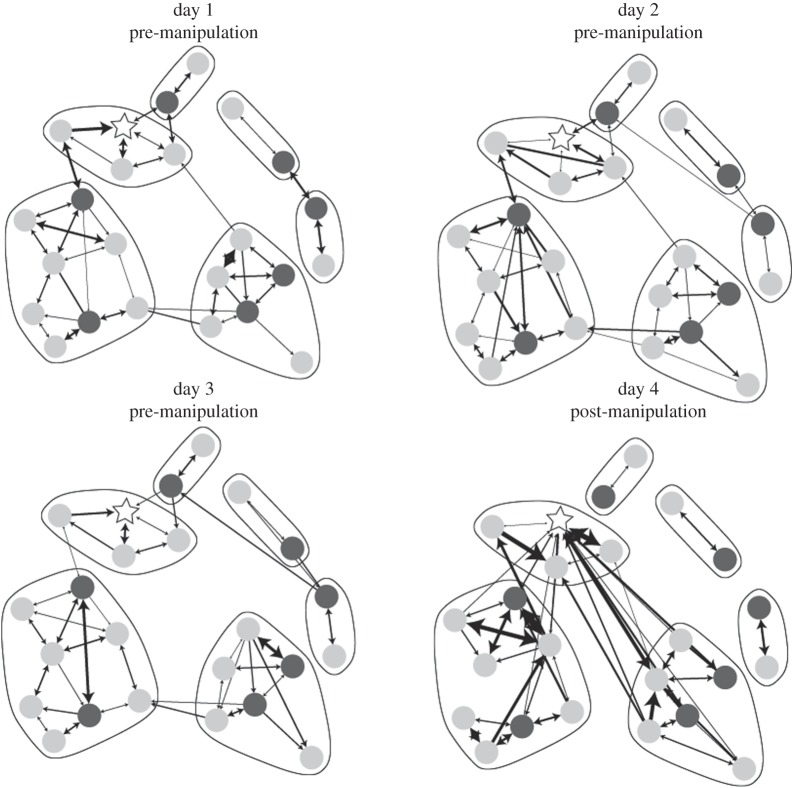
An example network is shown across the first 4 days of the experiment. Node placement is fixed across days and is based on a force-directed layout of the first day. Members in separate groups are outlined by solid lines, males are shown in dark grey, females in light grey and the male who had his territory augmented after the observation on day 3 is represented with a white star. Edge width is scaled to the rate of behavioural interactions between the individuals.

**Figure 2.**
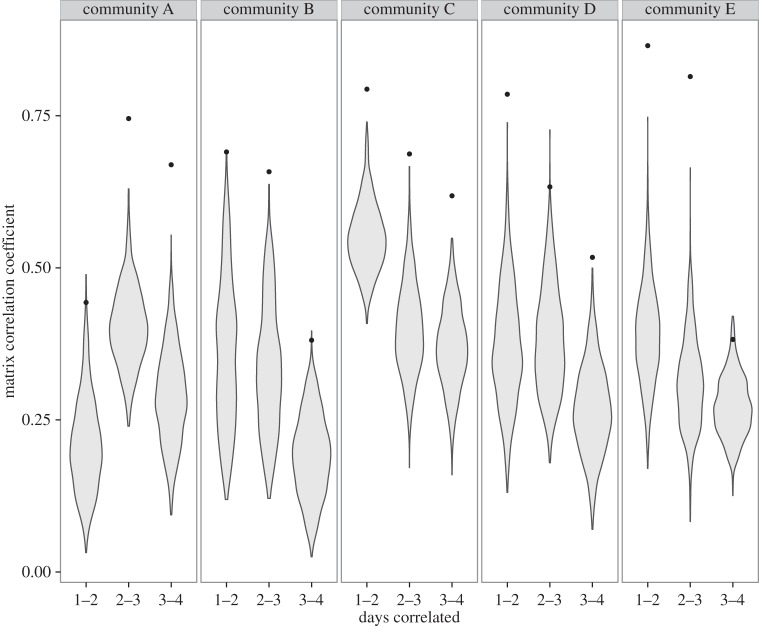
Violin plots showing the distribution of the randomized replicates with points showing the correlation coefficient of the real network. Overall matrix correlations between days decreased post-manipulation compared with pre-manipulation (day 3 compared with day 4, *p* = 0.0327). Social roles, community membership and interaction patterns were preserved across days compared to randomized networks. All comparisons between days 3 and 4 have an FDR-corrected *p* < 0.05, except for group E (*p* = 0.094).

In three of the five manipulated communities, the large heterospecific predator *Lepidolamprologus attenuatus* sp. ‘meeli’ (hereafter *L. attenuatus*) usurped augmented male territories, driving off or killing all resident *N. multifasciatus*. This predation event occurred at earliest by the fifth observation, causing severe disruptions to the social networks and restricting our network analyses to days 1–4, however full datasets are available on DRYAD. Community structure was highly modular, with the majority of interactions occurring within social groups (figure 1). Where intergroup interactions did occur, they were predominately border conflicts with nearest neighbour groups. Only males were observed to leave their territories and interact with members of groups that were not nearest neighbours. To assess the stability of individual associations over time, we correlated association matrices representing the networks across all seven pairwise, sequential time points. Prior to manipulations, we found strong correlations across time; all comparisons had an FDR-corrected *p*-value < 0.05 (figure 2). Between day 3 and day 4 (the first day after addition of extra shells), the correlation between days was significantly lower than the preceding two comparisons (planned contrasts, pre-manipulation versus post-manipulation: *Z* = 2.378, *p* = 0.0327). However, compared to the distribution of randomized networks, there was still a significant amount of non-random structure, such that post-manipulation all but group E had FDR-corrected *p*-values less than 0.05. This result demonstrates that although communities underwent significant changes after manipulation, they were not disrupted into complete randomness; the previous patterns of social interactions were maintained but to a lesser degree.

**Figure 3.**
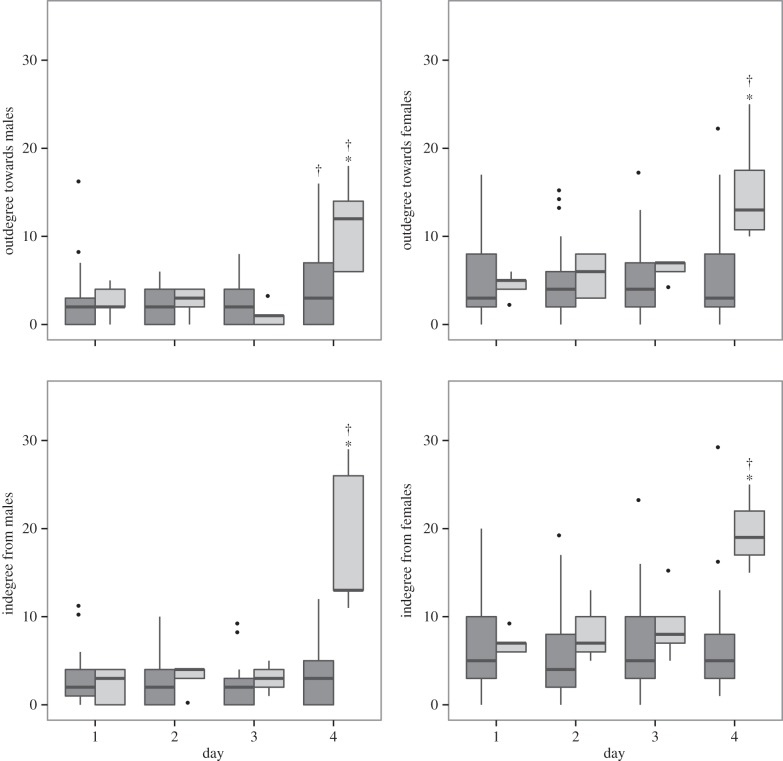
Boxplots of degree distributions (i.e. behavioural interactions), with manipulated males in light grey and non-manipulated males in dark grey. *p < 0.001 in the comparison between manipulated versus non-manipulated. ^†^*p* < 0.001 in the comparison between the first 3 days (prior to manipulation) and day 4 (post-manipulation) within treatment.

On the first day after experimental addition of extra shells to territories, there was a marked increase in the number of interactions the male territory owner had with other community members. We examined the degree densities of incoming (in-degree) and outgoing (out-degree) edges in the network as a function of sex and experimental manipulation (figure 3). We found support that both in-degree from males and females and out-degree towards males and females increased in manipulated males compared with unmanipulated males on day 4 (GLMM day × treatment. In-degree from males: *Z* = 4.992; *p* = 5.97 × 10^−7^, in-degree from females: *Z* = 6.713; *p* = 1.91 × 10^−11^, out-degree towards males: *Z* = 2.445; *p* = 0.015, out-degree towards females: *Z* = 5.674, *p* = 1.4 × 10^−8^). Furthermore, we found that manipulated males had significantly increased interactions in all four interaction types (in-degree from males or females, out-degree towards males or females) on day 4 compared with the days pre-manipulation (least-squares means: *p* of all models less than 0.0001), whereas non-manipulated males increased on day 4 only in their aggression towards other males (least-squares means: average of days 1–3 compared to day 4, *Z*-ratio = 2.827, *p* = 0.005).

### (b) Part II: reproductive advantages and ecological costs of increased territory attractiveness

**Figure 4.**
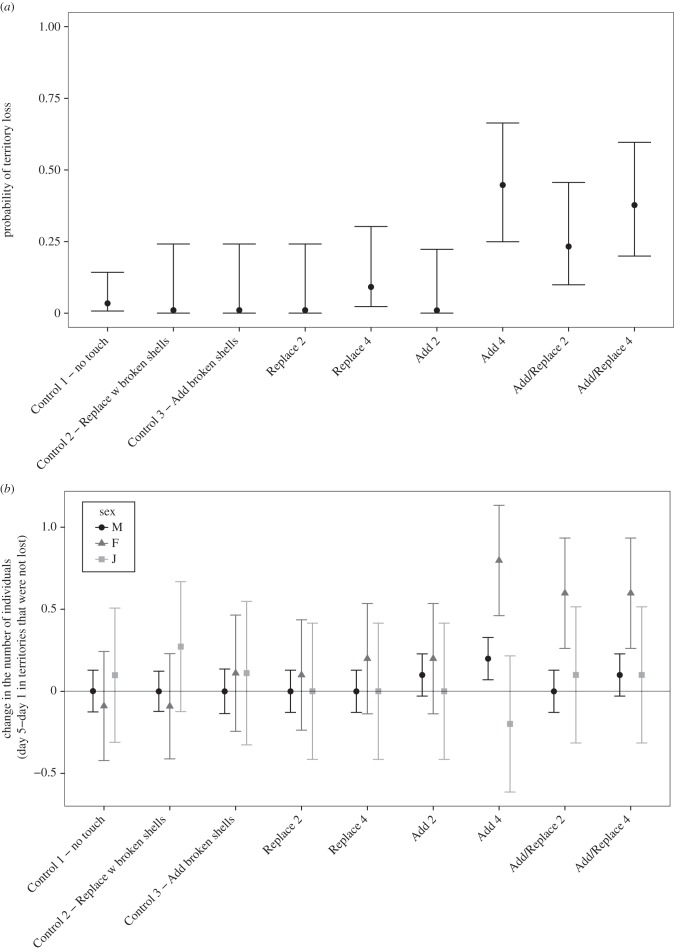
(*a*) Estimate and 95% CIs of the probability of territory loss for each treatment. (*b*) Mean and 95% CIs of the change in the number of males (black circles), females (grey triangles), and juveniles (grey squares). In Control 1, groups were left completely untouched, in Control 2 four broken shells were replaced with four different broken shells, and in Control 3 four broken shells were added to each group. Treatments Replace 2 and Replace 4 consisted of replacing two or four of the broken shells in the group with intact shells. Treatments Add 2 and Add 4 consisted of adding two or four intact shells to each group. Treatments Add/Replace 2 and Add/Replace 4 consisted of both replacing two or four shells *and* adding two or four shells to each group.

The frequency of territory loss was significantly different among treatment groups (figure 4*a*, binomial GLM *χ* = 37.261, d.f. = 9, *p* = 2.364 × 10^−5^). Post hoc analysis (nine planned comparisons, all treatments against appropriate controls, A4 against A2, R4 against R2, and AR4 against AR2) showed that three treatment groups (A4: *Z* = −2.45, *N* = 19, *p* = 0.032; AR2: *Z* = −3.10, *N* = 20, *p* = 0.009; AR4: *Z* = −3.410, *N* = 20, *p* = 0.006) had a significantly different rate of heterospecific takeover than groups in the control treatment. Furthermore, the comparisons between two or four shells revealed that there was a higher probability of territory loss in A4 as compared to A2 (*Z* = −2.52, *p* = 0.032). In total, 24 groups were evicted by heterospecific competitors. Of these, 22 were usurped by *L. attenuatus*, and two were usurped by *Neolamprologus brevis* (a slightly larger, non-group-living shell-dwelling species). Once territories were overtaken by *L. attenuatus*, resident *N. multifasciatus* were not seen again and the female *L. attenuatus* used *Neothauma* shells as a spawning site.

To examine changes in community structure, we analysed the subset of communities that were not taken over by heterospecifics. We subtracted the number of males and females present at the end of the experiment from the number in the first observation. The change in the number of females but not males varied significantly among treatment groups (figure 4*b*, ANOVA; males: *χ* = 15.767, d.f. = 9, *p* = 0.0719, females: *χ* = 51.716, d.f. = 9, *p* = 5.115 × 10^−8^). Post hoc analysis using the same nine planned comparisons detailed above showed that three treatment groups had significantly higher levels of female recruitment (figure 4*b*; A4: *Z* = −4.97, *N* = 10, *p* ≤ 0.0001; AR2: *Z* = −2.66, *N* = 15, *p* = 0.0174, AR4: *Z* = −3.522, *N* = 12, *p* = 0.0013). In addition, comparisons between two or four shells revealed that A4 had a higher level of female recruitment than A2 (*Z* = −3.522, *p* = 0.0013). It is notable that in the replacement treatments (R2 and R4), where broken shells were replaced with intact shells but no shells were added, there was no significant increase in either female attraction or risk of eviction.

## 4. Discussion

In this study, we examined the social and ecological sources of selection acting on an extended phenotype in the wild. By producing an ‘over-extended’ phenotype—the shell nest of the cichlid *N. multifasciatus—*we found evidence for positive sexual selection, in the form of increased access to mates, acting on augmented extended phenotypes (figure 4). Although we also found some evidence that territory augmentation leads to extra males joining these groups (figure 4*b*), the evidence was weak and only suggestive of a potential cost in the form of paternity loss to additional males. Similar to studies examining extended phenotypes in bowerbirds [10], we observed that benefits of increased attractiveness are offset by increased aggression from an extended network of community members (figure 3). Finally, our results suggest that there is strong ecological pressure in the form of increased risk of expulsion from augmented territories by heterospecific predators (figure 4). Taken together, these results reveal a suite of factors acting to shape the expression of an extended phenotype under natural conditions and provide insight into the evolution of extended and bodily sexual traits generally.

Extended phenotypes are likely to influence, and be influenced by, their broader social context. To test the extent of this influence, we used social network analyses to measure the local and global social impact of experimentally increased territory attractiveness. While global network structure was highly correlated within each community across days prior to manipulation, increasing the attractiveness of the extended phenotype significantly changed the network structure, such that network structures immediately prior to and following manipulation were not correlated. This result demonstrates that changes to the attributes of one node in a network (in this case the attractiveness of the male) can have far-reaching effects on the entire community structure (figures 1 and 2). The global change was driven by individual-level changes in responses to, and behaviours of, the newly attractive male (figure 3). Specifically, after territory augmentation affiliative interactions with females increased; males increased the number of courtship displays towards females and received more displays in return (figure 3*a*). In two cases, the increase in connection preceded a female leaving her current group and joining the group to which shells had been added. This is consistent with previous observations that group-living animals may frequently visit the territories of groups they later join [29,30]. These findings also suggest that network approaches can be used to predict the pattern of migration among groups prior to actual movement occurring, as well as allowing comparison between group membership preferences and the realization of these preferences as choice in restricted entry groups [31].

In contrast to the increased access to females afforded by larger territories, we also found strong social control acting to limit the expression of the extended phenotype. Significantly more aggression was directed towards manipulated males after territory augmentation (higher in-degree from males), and manipulated males responded with aggression of their own to neighbouring males (higher out-degree towards males; figure 3*b*). Unlike other species that can restore manipulated bowers to their original sizes after experimental manipulation [32], our study species is too small to move shells (thereby restoring original territory quality) and may therefore be unable to avoid increased aggression from neighbouring males. We observed two cases of rapid desertion of manipulated territories, and this may be one of the only ways individuals with territories in excess of their resource holding potential are able to avoid social punishment or increased predation risk. Social control of animal behaviour has been observed throughout taxa from social insects through to primates including humans [12], and may also operate for extended phenotypes [6]. Social punishment of males that bear phenotypes in excess of their own quality or resource-holding potential is known for bodily traits (e.g. house sparrow badges; [13,14]) and our results add to previous work suggesting this is also true for extended phenotypes (e.g. [10]). At an even broader scale, the interaction between the attributes of individuals and their social context is a topic of increasing research focus [33], and across digital, biological and sociological networks, and there is increasing research interest in the role of variation in the attributes of individual nodes shaping overall network structure [34]. The opportunity to manipulate extended phenotypes without unintended effects on other bodily traits allows for controlled manipulation of node attributes in animal social networks, and the approaches we use here provide a framework for understanding how this variation affects overall network structure and social dynamics under natural conditions.

The difficulty in measuring predation costs in natural experiments means that direct increases in predation associated with experimentally augmented sexual signals are rarely observed, despite a wealth of evidence that predation costs exist and shape sexual traits [33]. In this study, we found direct evidence that an increase in attractiveness of the sexual signal led to increased predation risk. In three of the five manipulated communities in part I, the larger piscivorous fish *L. attenuatus* took up residence in the experimentally more attractive *N. multifasciatus* territory, driving residents into surrounding territories or killing them directly. A follow-up experiment showed that augmenting the extended phenotype by adding extra shells almost doubled the likelihood of territory loss in the two most extreme manipulations (figure 4*a*). This increased risk was not associated with the actual manipulation of territories by experimenters, or by simple addition of more structure, as neither replacement nor addition of broken shells significantly increased the risk of predator takeover (figure 4*a*). This catastrophic ecological cost likely outweighs any benefit gained from possessing an attractive territory, and may be a primary source of counter-selection acting against increased expression of an extended phenotype.

Heterospecific takeover of experimentally manipulated territories is rarely reported in the literature, but the importance of predation in shaping territory size and quality has been suggested in both theoretical and correlational studies [15–17]. The paucity of reports may be a consequence of the tendency towards studying ornamental rather than resource-based extended phenotypes. Arbitrary ornamental traits, whether part of the body or extended phenotypes, by definition have little value outside that for the choosing female [35], and so are not expected to attract attention from heterospecifics. In contrast, many extended phenotypes serve multiple ecological roles, for example bird nest sites that also function as sexual signals [18,36], and in the current study, the shells used as breeding sites are also used by many other species [22,37]. It is therefore possible that heterospecific influence over resource-based extended phenotypes is widespread but poorly studied in comparison with ornamental extended phenotypes.

Given that adding new shells to a territory is risky, the process by which *N. multifasciatus* territories increase in size to reach the group sizes of 30 or more individuals and more than 100 shells [21] is still unclear. Our own field observations, as well as previous experiments, suggest that female group members are highly resistant to joining attempts by other females [19], a pattern of intrasexual aggression also found in many birds [38,39]. By increasing the resource pool within the territory, potential group joiners may be able to find shelter to escape aggression and integrate more easily into the new group [40]. It is possible that in resource-based extended phenotypes, males extend the trait just beyond immediate requirements in an attempt to attract new females, but not above a level that becomes too attractive to competitors that may also use the resource. This interpretation is supported by our findings that neither adding broken shells (which are not used as spawning or shelter sites), nor adding or replacing only two intact shells significantly increased usurpation risk (figure 4).

We suggest that social and ecological costs acting on extended phenotypes may play a significant role in the evolution of sexual traits. However, because predation occurs too rarely to be readily observed within the experimental time frame of most studies, and because social costs may be mild or otherwise difficult to measure, these two processes may frequently be obscured by stronger proximate costs associated with bodily trait development and maintenance. Here, we experimentally avoid the effects of these proximate factors by manipulating an extended phenotype without directly producing collateral effects on other traits. This provides the opportunity to clearly distinguish and examine the sources of selection acting on sexual traits in the wild and determine how sources of selection on extended phenotypes differ in strength and form from those reported for bodily traits.

## Supplementary Material

Supplementary file

## Supplementary Material

Photo of Neolamprologus multifasciatus in Lake Tanganyika

## Supplementary Material

Photo of Neolamprologus multifasciatus in Lake Tanganyika

## Data accessibility

The datasets supporting this article have been uploaded to the Dryad data repository at: http://dx.doi.org/10.5061/dryad.61vk4.

## References

[1] Andersson M 1994 Sexual selection. Princeton, NJ: Princeton University Press.

[2] Arnold SJ 1983 Sexual selection: the interface of theory and empiricism. In Mate choice (ed. Bateson PPG), pp. 67–107. Cambridge, UK: Cambridge University Press.

[3] Shuster SM, Wade MJ 2003 Mating systems and strategies. Princeton, NJ: Princeton Universiy Press.

[4] Jordan LA, Brooks RC 2010 The lifetime costs of increased male reproductive effort: courtship, copulation and the Coolidge effect. J. Evol. Biol. 23, 2403–2409. (10.1111/J.1420-9101.2010.02104.X) 20825547

[5] Nonacs P, Blumstein DT 2010 Predation risk and behavioral life history. In Evolutionary behavioral ecology (eds Westneat DF, Fox CW), pp. 207–221. London, UK: Blackwell Scientific Press.

[6] Schaedelin FC, Taborsky M 2009 Extended phenotypes as signals. Biol. Rev. 84, 293–313. (10.1111/J.1469-185x.2008.00075.X) 19382933

[7] Borgia G 1995 Complex male display and female choice in the spotted bowerbird: specialized functions for different bower decorations. Anim. Behav. 49, 1291–1301. (10.1006/Anbe.1995.0161)

[8] Diamond J 1986 Animal art: variation in bower decorating style among male bowerbirds *Amblyornis inornatus*. Proc. Natl Acad. Sci. USA 83, 3042–3046. (10.1073/Pnas.83.9.3042) 16593691 PMC323443

[9] Borgia G 1985 Bower quality, number of decorations and mating success of male satin bowerbirds (*Ptilonorhynchus violaceus*): an experimental analysis. Anim. Behav. 33, 266–271. (10.1016/S0003-3472(85)80140-8)

[10] Borgia G 1985 Bower destruction and sexual competition in the satin bowerbird (*Ptilonorhynchus violaceus*). Behav. Ecol. Sociobiol. 18, 91–100. (10.1007/Bf00299037)

[11] Miles AJ, Madden JR 2002 Bower location by the spotted bowerbird (*Chlamydera maculata*). EMU 102, 187–193. (10.1021/Mu00039)

[12] Gardner A, Griffin AS, West SA 2010 Altruism and cooperation. In Evolutionary behavioral ecology (eds Westneat DF, Fox CW), pp. 308–326. Oxford, UK: Oxford University Press.

[13] Rohwer S 1977 Status signaling in harris sparrows: some experiments in deception. Behaviour 61, 106 (10.1163/156853977X00504)

[14] Moller AP 1987 Variation in badge size in male house sparrows *Passer domesticus*: evidence for status signaling. Anim. Behav. 35, 1637–1644. (10.1016/S0003-3472(87)80056-8)

[15] Taylor RJ 1988 Territory size and location in animals with refuges: influence of predation risk. Evol. Ecol. 2, 95–101. (10.1007/BF02067270)

[16] Magnhagen C 1991 Predation risk as a cost of reproduction. Trends Ecol. Evol. 6 183–186. (10.1016/0169-5347(91)90210-O) 21232452

[17] Candolin U, Voigt H-R 2001 Correlation between male size and territory quality: consequence of male competition or predation susceptibility? Oikos 95, 225–230. (10.1034/j.1600-0706.2001.950204.x)

[18] Mainwaring MC, Hartley IR, Lambrechts MM, Deeming DC 2014 The design and function of birds’ nests. Ecol. Evol. 4, 3909–3928. (10.1002/ece3.1054) 25505520 PMC4242575

[19] Schradin C, Lamprecht J 2000 Female-biased immigration and male peace-keeping in groups of the shell-dwelling cichlid fish *Neolamprologus multifasciatus*. Behav. Ecol. Sociobiol. 48, 236–242. (10.1007/S002650000228)

[20] Schradin C, Lamprecht J 2002 Causes of female emigration in the group-living cichlid fish *Neolamprologus multifasciatus*. Ethology 108, 237–248. (10.1046/J.1439-0310.2002.00775.X)

[21] Kohler U 1998 Zur Struktur und Evolution des Sozialsystems von *Neolamprologus multifasciatus* (Cichlidae, Pisces), dem kleinsten Schneckenbuntbarsch des Tanganjikasees. Aachen, Germany: Shaker Verlag.

[22] Kawanabe H, Hori M, Nagoshi M 1997 Fish communities in Lake Tanganyika. Kyoto, Japan: Kyoto University Press.

[23] Csardi G, Nepusz T 2006 The igraph software package for complex network research. *InterJournal, Complex Systems* 1695. See http://igraph.org.

[24] James R, Croft DP, Krause J 2009 Potential banana skins in animal social network analysis. Behav. Ecol. Sociobiol. 63, 989–997. (10.1007/s00265-009-0742-5)

[25] Hobson EA, Avery ML, Wright TF 2013 An analytical framework for quantifying and testing patterns of temporal dynamics in social networks. Anim. Behav. 85, 83–96. (10.1016/j.anbehav.2012.10.010)

[26] Pons P, Latapy M 2005 Walktrap algorithm. Computing communities in large networks using random walks (long version). (http://arxiv.org/abs/physics/0512106)

[27] Elston D, Moss R, Boulinier T, Arrowsmith C, Lambin X 2001 Analysis of aggregation, a worked example: numbers of ticks on red grouse chicks. Parasitology 122, 563–569. (10.1017/S0031182001007740) 11393830

[28] Gelman A, Jakulin A, Pittau MG, Su Y-S 2008 A weakly informative default prior distribution for logistic and other regression models. Ann. Appl. Stat. 2, 1360–1383. (10.1214/08-AOAS191)

[29] Bergmüller R, Heg D, Taborsky M 2005 Helpers in a cooperatively breeding cichlid stay and pay or disperse and breed, depending on ecological constraints. Proc. R. Soc. B 272, 325–331. (10.1098/rspb.2004.2960) PMC1634971 15705559

[30] Jordan LA, Wong MYL, Balshine SS 2010 The effects of familiarity and social hierarchy on group membership decisions in a social fish. Biol. Lett. 6, 301–303. (10.1098/rsbl.2009.0732) 20007168 PMC2880034

[31] Jordan LA, Avolio C, Herbert-Read JE, Krause J, Rubenstein DI, Ward AJW 2010 Group structure in a restricted entry system is mediated by both resident and joiner preferences. Behav. Ecol. Sociobiol. 64, 1099–1106. (10.1007/s00265-010-0924-1)

[32] Schaedelin FC, Taborsky M 2006 Mating craters of *Cyathopharynx furcifer* (Cichlidae) are individually specific, extended phenotypes. Anim. Behav. 72, 753–761. (10.1016/J.Anbehav.2005.11.028)

[33] Kurvers RH, Krause J, Croft DP, Wilson AD, Wolf M 2014 The evolutionary and ecological consequences of animal social networks: emerging issues. Trends Ecol. Evol. 29, 326–335. (10.1016/j.tree.2014.04.002) 24792356

[34] Lawyer G 2015 Understanding the influence of all nodes in a network. Sci. Rep. 5, 8665 (10.1038/srep08665) 25727453 PMC4345333

[35] Andersson M, Simmons LW 2006 Sexual selection and mate choice. Trends Ecol. Evol. 21, 296–302. (10.1016/j.tree.2006.03.015) 16769428

[36] Moreno J 2012 Avian nests and nest-building as signals. Avian Biol. Res. 5, 238–251. (10.3184/175815512X13534385822786)

[37] Mitchell JS, Ocana SW, Taborsky M 2014 Male and female shell-brooding cichlids prefer different shell characteristics. Anim. Behav. 98, 131–137. (10.1016/j.anbehav.2014.10.004)

[38] Slagsvold T, Amundsen T, Dale S, Lampe H 1992 Female–female aggression explains polyterritoriality in male pied flycatchers. Anim. Behav. 43, 397–407. (10.1016/S0003-3472(05)80100-9)

[39] Iwasa Y, Harada Y 1998 Female mate preference to maximize paternal care. II. Female competition leads to monogamy. Am. Nat. 151, 367–382. (10.1086/286125) 18811327

[40] Bergmuller R, Heg D, Peer K, Taborsky M 2005 Extended safe havens and between-group dispersal of helpers in a cooperatively breeding cichlid. Behaviour 142, 1643–1667. (10.1163/156853905774831800)

