# Supplementary material for: The social and ecological costs of an ‘over-extended' phenotype
Source: Proc Biol Sci. 2016 Jan 13;283(1822):20152359. doi: 10.1098/rspb.2015.2359 (PMC4721094; doi:10.1098/rspb.2015.2359)
Supplement: Supplementary file [file rspb20152359supp1.pdf]

Supplementary Materials

FOR

The social and ecological costs of an 'over-extended' phenotype

Lyndon Alexander Jordan[1, 2]*, Sean M. Maguire[1], Hans A. Hofmann[1, 3], Masanori Kohda[2]

1. Department of Integrative Biology, The University of Texas at Austin, One University Station, Austin TX 78712, USA

2. Laboratory of Animal Sociology, Department of Biology and Geosciences, Graduate School of Science, Osaka City University, Osaka 558-8585, Japan

3. Institute for Cellular and Molecular Biology, Institute for Neuroscience, The University of Texas at Austin

*Author for correspondence: lyndonjordan@gmail.com

Present address: Department or Collective Behaviour, Max Planck Institute for Ornithology, Konstanz

<u>SUPPLEMENTARY MATERIALS</u>

SM1. Natural History of study species

The cichlid fish *Neolamprologus multifasciatus* is endemic to Lake Tanganyika (Africa), and one of the world's smallest cichlids, if not the smallest. There is no pronounced sexual dimorphism in coloration, but males reach a larger maximum size (30 mm SL) than do females (20 mm SL) [1, 2] .The colour pattern consists of pale vertical bars on a light brown background and is specific to the individual, allowing for identification based on pattern. This species lives in stable social groups on beds of fossilized *Neothauma* gastropod shells [1], which it uses both for shelter from predators and as brood chambers in which the smaller females deposit eggs [2]. The shells in these beds are not used exclusively by *N. multifasciatus*, being also used for breeding and shelter by numerous other cichlids, including those in the genera *Neolamprologus*, *Telmatochromis*, *Lepidiolamprologus* and *Xenotilapia*, some of which we observed taking temporary shelter in *N. multifasciatus* territories. Each group occupies a small depression in the lake bottom containing a number of empty shells that have been excavated from the sand, and is generally bordered by one or more neighbouring territories of other groups. The border between territories is typically demarcated by a ridge of sand that is continually added to by group members taking mouthfuls of excavated sediment and depositing it on the territory boundaries. Large communities of *N. multifasciatus* thus have the appearance of a pockmarked and cratered landscape of depressions and ridges, and may extend for hundreds of metres in all directions. Groups commonly contain several reproductive males and females and their offspring, often from several clutches. Breeding males may be siblings, and breeding females within groups are occasionally related to the breeding males [2]. However, microsatellite analysis detected unrelated individuals within groups, demonstrating that dispersion among groups occurs under natural conditions. Individuals feed on plankton in the water column above the territory, and almost never venture outside of their immediate territory [2].

Previous research has shown that the number of shells in a male's nest directly influences the likelihood of new females joining his group, and the number of shells in a group increases with the number of individuals [3, 4]. Females in a group are aggressive to other females who attempt to join groups and females also compete within groups for access to shells and defend sub-territories against other females [3, 4]. While nest maintenance is shared among both male and female group members, previous experiments suggest that males are primarily responsible for addition of extra shells and receive the greatest social benefit, in terms of increased access to mates, from increasing nest size [3, 4]. Despite the fact that thousands of shells cover the lake floor in some places, covered by only a thin layer of sediment, intact and unoccupied shells may still prove a valuable resource for a number of reasons. First, shells that are already excavated are almost always within an established territory of *N. multifasciatus* or a larger shell-dwelling heterospecific, and therefore defended by residents. Second, the density of predators of *N. multifasciatus* in the

lake is very high, placing severe limitations on the ability of individuals to safely excavate new shells, even in areas directly adjacent to existing territories [2, 3]. In any case, *N. multifasciatus* cannot steal shells from existing territories or undefended patches because they are too small to physically move *Neothauma* shells. Importantly for our experiment, this also means *N. multifasciatus* cannot remove shells that have been experimentally added to territories. Because of this inability to easily modify the number of shells in nests, we did not conduct any experiments where shells were removed from territories. In pilot tests, LAJ observed that removing shells from wild groups caused some individuals being left without sufficient shelter and potentially subject to increased predation, and for ethical reasons this type of experiment was not carried out.

Although it is apparently dangerous, we did directly observe the founding of two new territories by males, who over the course of three days uncovered three or four shells. These males moved a short distance away from their original territory and began to uncover one or a few shells from the sediment by taking mouthfuls of sand away from the excavation site, and quickly swam back to their group territory when any disturbance occurred (pers. obs.). One of these males was joined by a female (group A3 in the networks experiment) and established a new territory, while the other was observed to build the territory, but was not seen again and may have been lost to predation.

In our experiments, we frequently observed the larger piscivorous cichlid *Lepidiolamprologus attenuatus* sp. meeli taking up residence and breeding in the territories of *N. multifasciatus*. The reasons the larger *L. attenuatus* choose to take over established territories rather than build their own nests remain unknown. It is possible that *N. multifasciatus* are one of the few cichlids small enough to adequately clear the sediment from inside *Neothauma* shells and thus render them suitable for use as breeding shelters by larger heterospecifics like *L. attenuatus.* If true, this would suggest an interesting interrelationship between the two species that warrants further attention.

In one case, an individual *Neolamprologus brevis* co-existed within the territory, without displacing the resident *N. multifasciatus*, until the end of the observation period. In a further two cases, non-piscivorous *Xenotilapia* species were observed within the territories of *N. multifasciatus*, but were not observed within territories in subsequent observations. These were not scored as evictions.

SM2. Social network analysis of increased territory attractiveness

Five extended social communities comprised of either six or seven distinct groups were identified. Each group in these communities had social connections (e.g. border conflicts, aggressive displays, courtship displays) with at least one other group in the community and no connections with any group outside the community. One group in each community, always containing two males and between two and four females, was haphazardly chosen to undergo experimental manipulation of shell number within the territory. For three subsequent days, a single SCUBA diver

(LAJ) conducted focal behavioural observations to measure the baseline social network structure of the community. Each fish (mean +/- s.e. number of fish per group = 3.55, +/- 1.46, n = 34; per community = 23.4 +/- 1.14, n = 5) was observed for five minutes and all interactions with other group members were recorded. We chose to use direct behavioural observations rather than affiliation to provide a more robust and biologically meaningful measurement of social relationships.

Individual fish were identified by the pattern of vertical bars on their flanks. Observations were recorded with a Sony G12 digital camera to later confirm the identity of individuals that could not be immediately recognized, but the site fidelity of individuals greatly reduced confusion about individual identity as fish only rarely moved more than 30 cm from their territory. Immediately after the third observation, six intact, empty *Neothauma* shells (35 – 45 mm diameter) were added to the predefined experimental group, and after 24 hours behavioural observations were taken for a further four days. To investigate how the manipulation affected the stability of the group we used the quadratic assignment procedure (QAP), a permutation technique that swaps node labels among different members of the social network. This is used to create a non-parametric null distribution to facilitate statistical testing on social networks, where observations are inherently non-independent. However, if QAP is used without any restrictions on which node labels can be swapped, it can lead to very unrealistic and non-conservative null distributions. This is because of inherent structure in animal social networks, for example males may tend to have particular patterns of interactions that are fundamentally different from females. To create a more realistic and conservative testing procedure, we restricted the node label swapping within sex and between members of densely connected groups, as recommended by James et al [5]. We identified members of densely connected groups in an unbiased way using the Walktrap algorithm [6] implemented in the R package igraph. This algorithm identifies densely connected groups using short random walks along the network connections, based on the fact that members of the same group tend to interact frequently and within a short interaction distance of one-another. In addition to this we used several other community detection methods to identify groups, including label propagation and edge-betweeness and found comparable results (data not shown). Overall we found that using this restricted modified version of the QAP gave us more conservative estimates than using random label swapping.

SM3. Analysis of the effect of distance to the manipulated group and territory area

We measured the distances among the centres of each territory to the centres of every other territory within each community, as well as the total area occupied by each group, using scaled overhead photographs taken in the lake and analysed in imageJ. We used a central point in the territory for measurements because individuals move freely within the area they occupy. We then tested whether the response to males that had been made experimentally more attractive was

dependent on distance from the manipulated group or territory size. The group average in- and out-degree towards or from the manipulated males were then used as response variables in models that included distance to the manipulated group and territory size as covariates, for each of the first four days of our observations (Days 1-3 are pre-manipulation, Day 4 is post-manipulation). We used a linear mixed model with a random effect for each observation of each community (community:day, n = 240 group averages, 33 random effect groups). Models were run separately for females and males. We looked for an interaction between day and distance, and day and size, which would indicate that the correlation between distance or size and the association metric (in- or out-degree for males or females) changed after the manipulation. Territory size was not significantly correlated to its distance to the manipulated territory ($R^2 = 0.027$, $p = 0.788$).

We found evidence that interactions were affected by the distance to the manipulated territory on day 4 (Supplementary Figure S1, day: distance). The interactions (out-degree) of manipulated males towards unmanipulated males decreased with distance ($t =- 2.070$, $p = 0.0417$; Fig S1A), as did the out-degree of manipulated males towards females ($t = -3.215$, $p = 0.001776$; Fig S1B). Similarly, for in-degree the interactions from unmanipulated males towards manipulated males decreased with distance to the manipulated male's group ($t = -2.500$, $p = 0.01451$; Fig S1C), as did the in-degree of females towards manipulated males ($t = -3.634$ $p = 0.00045$, Fig S1D).

Territory size was also a significant predictor of most association metrics examined (Supplementary Figure S2). The interactions (out-degree) of manipulated males towards unmanipulated males did not increase with group size ($t = 1.612$, $p = 0.1110$; Fig S2A), whereas the out-degree of manipulated males towards females increased with the size of the group those females were in ($t = 3.563$ $p = 0.000574$; Fig S2B). Similarly, the interactions (in-degree) of unmanipulated males towards manipulated males increased with the size of the unmanipulated males' group ($t = 4.442$, $p = 2.9 \times 10^{-5}$; Fig S2C), as did the in-degree of females towards manipulated males ($t = 2.800$, $p = 0.00617$, Fig S2D).

SM4. Alternate analysis of the probability of heterospecific territory takeover using pooled controls
In an alternative analysis of the probability of territory takeover (*cf.* Figure 4A in main text), we compared all treatments to the pooled controls (i.e. combining C1, C2 and C3). We found very similar results to the planned contrasts presented in the main text. The frequency of territory loss was significantly different among treatment groups (Binomial GLM $\chi = 39.793$, df = 7, $P = 1.379 \times 10^{-6}$). Post-hoc analysis (all treatments against pooled controls, A4 against A2, R4 against R2, and AR4 against AR2) showed that three treatment groups (A4: Z = 3.79, N = 19, P = 0.0014; AR2: Z = 2.53, N = 20, P = 0.0285; AR4: Z = 3.511, N = 20, P = 0.002) had a significantly different rate of heterospecific takeover than groups in the control treatment. Furthermore, the comparisons

between two or four shells revealed that there was a higher probability of territory loss in A4 as compared to A2 ($Z = -2.49$, $P = 0.0285$).

SM5. Alternative social network construction and analysis

In the main text we presented social networks where edges represented the sum of all behavioural interactions directed from one individual towards another. In an alternate analysis we divided the behaviours into two sets: either displays or direct behavioural interactions (chases, rams, bites). Displays were the most common type of interaction observed, while direct interactions were less common. Same-sex displays (i.e. male-to-male and female-to-female) encompassed puffed throat, aggressive postures, and head shaking, and were scored as aggressive interactions [7]. Similarly, all same-sex bites, rams, and chases were scored as aggressive. Male-female and female-male quivers, hook (or 'J' displays), and soft touches were scored as affiliative [7]. We repeated the modified quadratic assignment procedure (QAP) and the analysis of degree distributions in the same way described in the main text (Figures 2 and 3). The results of this analysis are presented below in sections SM6 and SM7.

SM6. Network analysis of detailed behavioural interactions

For display behaviour (supplementary figure S3A) we found very similar results to the summed behaviour presented in the main text. The lowest correlations were found between the networks on day three and day four (the comparison between pre- and post-manipulation) indicating that the manipulation altered the social network. However, the alteration of the network post-manipulation did not disrupt the pattern of social interactions completely because the correlations were still stronger than the random expectation generated by the QAP (P-values of all comparisons < 0.05).

The analysis of chase, bite and soft-touch behaviour (supplementary figure S3B) showed a different pattern, mainly because the networks created using these measures were far more sparse. Comparisons between networks on subsequent days were often not higher than the random expectation generated by the QAP procedure (see supplementary figure S3B, comparisons marked with an asterisk have a P-value < 0.05). This indicates that there is not as much structure in these networks that is consistent across days, probably because of the relative rarity of these direct behaviours. We are hesitant to draw any further conclusions from this type of underpowered analysis, but can say that there is no clear or consistent effect of the day four manipulation on these interaction networks based on physical behavioural interactions.

SM7. Degree Distributions

For display behaviours (supplementary figure S4) our results were very similar to the results presented in the main text. We found support that both in-degree from males and females and out-degree towards males and females increased in manipulated males compared to unmanipulated

males on day 4 (GLMM day*treatment. In-degree from males: Z = 4.390; P = 1.14 x10^{-5}, in-degree from females: Z =6.135; P = 8.5x10^{-10}, out-degree towards males: Z = 3.195; P = 0.0014, out-degree towards females: Z = 5.556; P = 2.76x10^{-8}). Furthermore, we found that manipulated males had significantly increased displays in all four interaction types (in-degree from males or females, out-degree towards males or females) on day four compared to the days pre-manipulation (least-squares means: *P* of all models < 0.0001). In contrast to the results presented in the main text, we did not find any significant changes among unmanipulated males post-manipulation in any of these interaction types.

For chase, bite and soft-touch behaviours (supplementary figure S5) our results were qualitatively different from the results presented in the main text. We found that in-degree from males and females, but not out-degree, increased post manipulation in the manipulated males (GLMM day*treatment. In-degree from males: Z = 3.139; P = 0.00170, in-degree from females: Z = 5.167; P = 2.37x10^{-7}, out-degree towards males: Z = 0.829; P = 0.40703, out-degree towards females: Z = 1.737; P = 0.0824). Furthermore we found that, although there was no significant difference between manipulated and unmanipulated males on day four, both manipulated and unmanipulated males did increase their out-degree over the pre-manipulation time points (least-squares means of day 4 compared with the average of days 1-3, manipulated males: Z-ratio = 3.503894; P < 0.001, unmanipulated males: Z-ratio = 3.936455; P < 0.001).

SM8. Alternative analysis of the probability of heterospecific take-over and demographics that includes all observation time points.

In the main text we included an analysis of the end points of the experimental period (observation 5 – observation 1). Here we present data from all five observation days using a repeated measures design. The conclusions of this analysis are qualitatively identical to the results presented in the main text. To investigate the overall effect of treatment on the frequency of territory takeover by heterospecifics we performed non-parametric Kruskal-Wallis tests, with Mann-Whitney U post-hoc tests to test for significant pairwise contrasts against control treatments. Changes in the number of male, female, or juvenile group members were analysed using repeated measures general linear models in SPSS 22. Time (T1 - T5) was the repeated measure within each group, with treatment as a fixed factor and the original number of shells in each territory included as a random covariate in the model. Data were tested and found to satisfy the assumption of homoscedasticity for linear models, however the assumption of sphericity was not met in all cases and so Greenhouse-Geisser tests are reported [8].

Many experimental groups were taken over by heterospecific competitors, resulting in complete loss of all group members. Because of this, two separate analyses were conducted for each response variable. The first analysis included all groups that were successfully tracked until the final observation. For the second analysis, groups that were usurped by heterospecific competitors

were removed from the analysis. Because this resulted in unequal sample sizes among treatments, sample sizes were balanced by randomly removing groups from over-represented treatments so that N = 10 for all treatments. Both sets of analysis are reported below (Supplementary Figures 6 and 7).

The frequency of territory loss was significantly different among treatment groups (Kruskal-Wallis χ = 47.33 df = 8 P < 0.001). Holm's sequential Bonferroni [9] corrected pairwise Mann-Whitney U tests against the control treatment showed that treatment groups (A4: U = 110, N = 20, P = 0.001 two-tailed, α = 0.017; AR2: U = 150, N = 20, P = 0.018 two-tailed, α = 0.05; AR4: U = 120, N = 20, P = 0.002 two-tailed, α = 0.025) had a significantly different rate of heterospecific takeover than groups in the control treatment. In total 24 groups were evicted by heterospecific competitors. Of these, 22 were usurped by *L. attenuatus*, and 2 were usurped by *Neolamprologus brevis* (a slightly larger, non group-living shell-dwelling species). Once territories were overtaken by *L. attenuatus*, resident *N. multifasciatus* were not seen again, and the female *L. attenuatus* used *Neothauma* shells as a spawning site. In one case, an individual *N. brevis* co-existed within the territory, without displacing the resident *N. multifasciatus,* until the end of the observation period. In a further two cases, non-piscivorous *Xenotilapia* species were observed within the territories of *N. multifasciatus*, but were not observed within territories in subsequent observations. These were not scored as evictions.

When all groups were included in analyses, there were no differences in the number of males (Repeated measures GLM: Greenhouse-Geisser F_{df} = 1.527 $_{19.0}$, P = 0.073; Figure S5), females (Greenhouse-Geisser F_{df} = 0.517 $_{19.1}$, P = 0.995) or juveniles (Greenhouse-Geisser F_{df} = 1.505 $_{20.2}$, P = 0.074) between treatment groups over the course of the observational period. However, the general trend for males and juveniles was a reduction in number over the observation period due to the loss of members in groups that were taken over by conspecifics and subsequently evicted. When usurped groups were excluded from the analysis, significant differences were detected among treatment groups in the number of females over time (Greenhouse-Geisser F_{df} = 2.676 $_{17.7}$, P = 0.001), but no differences were found in the number of males (Greenhouse-Geisser F_{df} = 0.980 $_{13.1}$, P = 0.448) or juveniles (Greenhouse-Geisser F_{df} = 0.980 $_{18.9}$, P = 0.486; Figure S6). There was no interaction between treatment and the initial number of shells in each group for any response variable (Males: Greenhouse-Geisser F_{df} = 0.236 $_{1.6}$, P = 0.745; Females: Greenhouse-Geisser F_{df} = 0.948 $_{2.2}$, P = 0.397; Juveniles: Greenhouse-Geisser F_{df} = 0.520 $_{2.4}$, P = 0.626). FDR corrected pairwise t-tests were conducted between the control treatment and the three treatment groups most likely to contribute to the significant overall effect (A4, AR2, and AR4). All three of these groups had significantly higher female joining than control groups (A4: $t,_{df}$ = 2.91,$_{18}$ P = 0.009 α = 0.025; AR2: $t,_{df}$ = 2.23,$_{18}$ P = 0.032 α = 0.05; AR4: $t,_{df}$ = 3.13,$_{18}$ P = 0.006 α = 0.017).

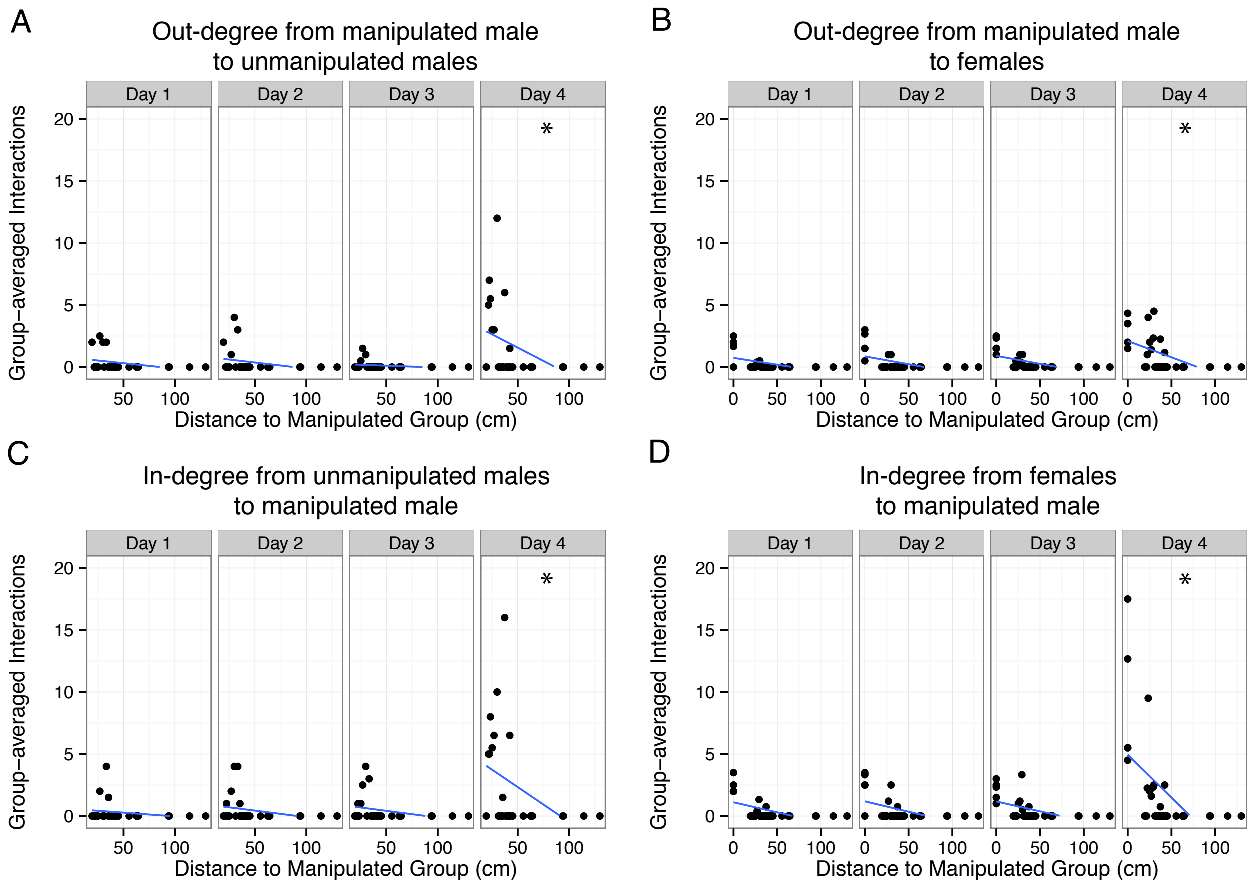

Supplementary Figure S1. Correlations between distance to the manipulated group and group averaged interaction metrics. Group averaged interactions of A) Manipulated males directed towards unmanipulated males, B) Manipulated males directed towards females, C) Unmanipulated males directed towards manipulated males, D) Females directed towards manipulated males. Asterisks indicate a significant day x distance interaction effect at $p < 0.05$).

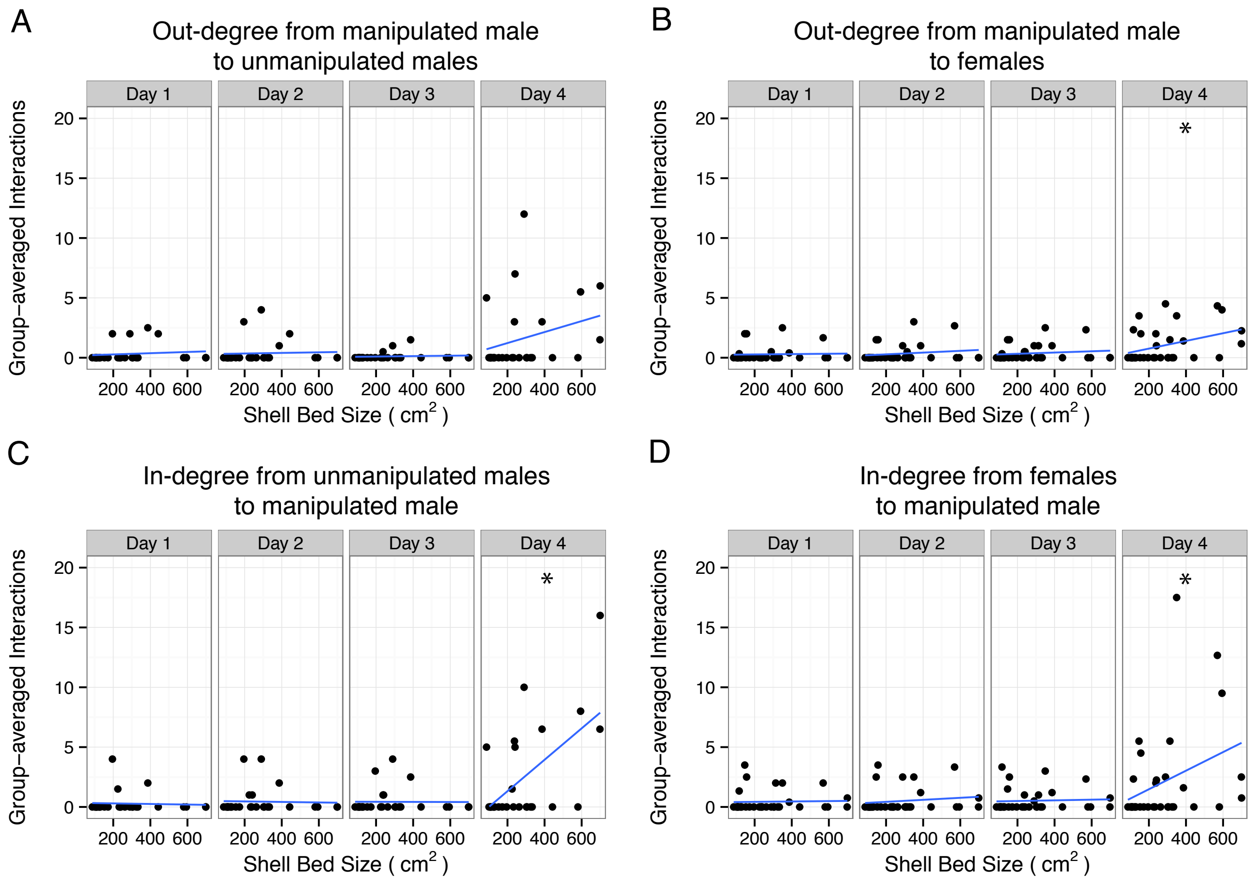

Supplementary Figure S2. Correlations between territory size of the group and group averaged interaction metrics. Group averaged interactions of A) Manipulated males directed towards unmanipulated males, B) Manipulated males directed towards females, C) Unmanipulated males directed towards manipulated males, D) Females directed towards manipulated males. Asterisks indicate a significant day x size interaction effect at $p < 0.05$).

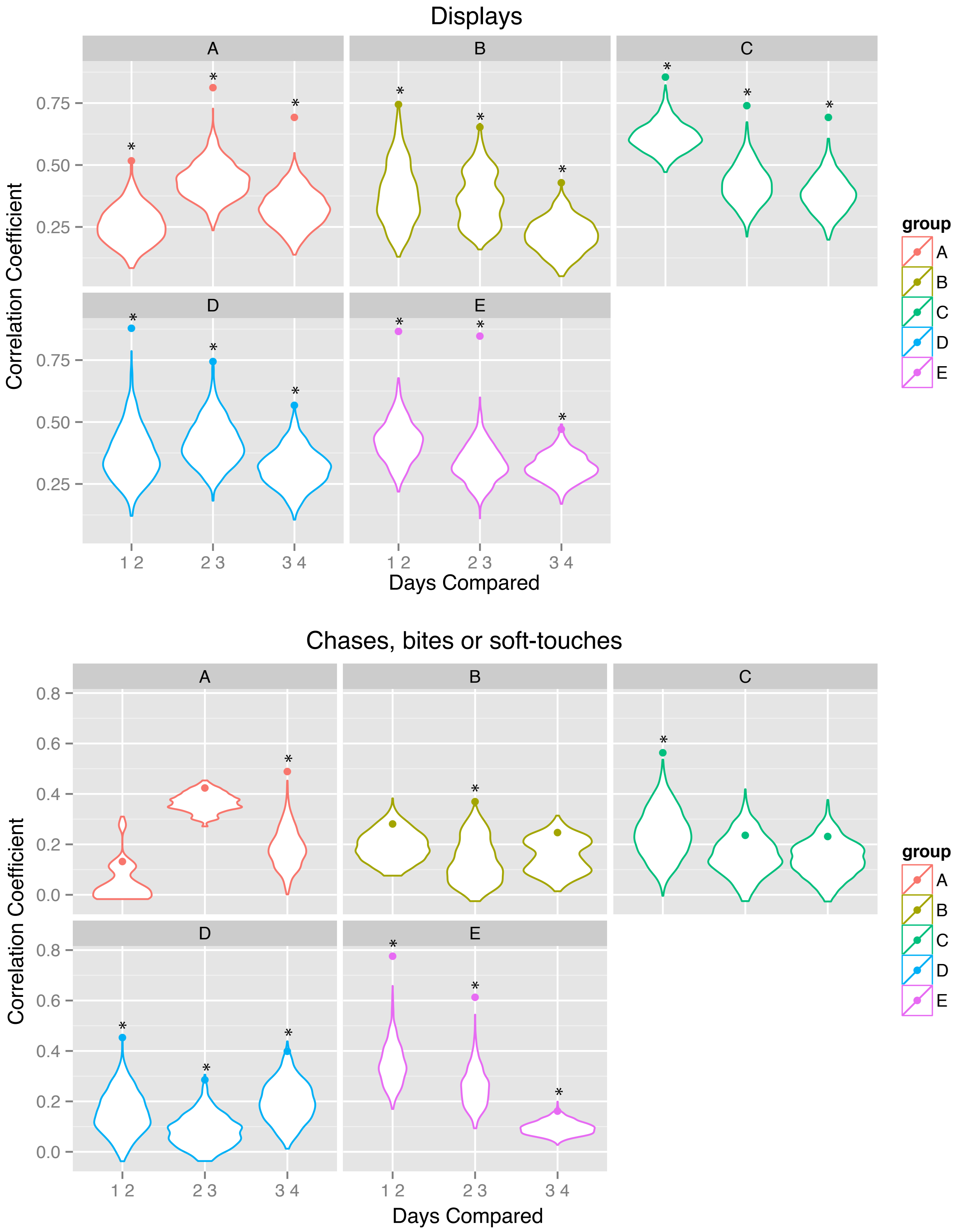

Supplementary Figure S3. QAP assignment procedure on networks comprised of A) displays and B) chases, bites and soft-touches. Violin plots show the distribution of the randomized replicates while points show the correlation coefficient of the real networks. Comparisons with P less than 0.05 are indicated with an asterisk.

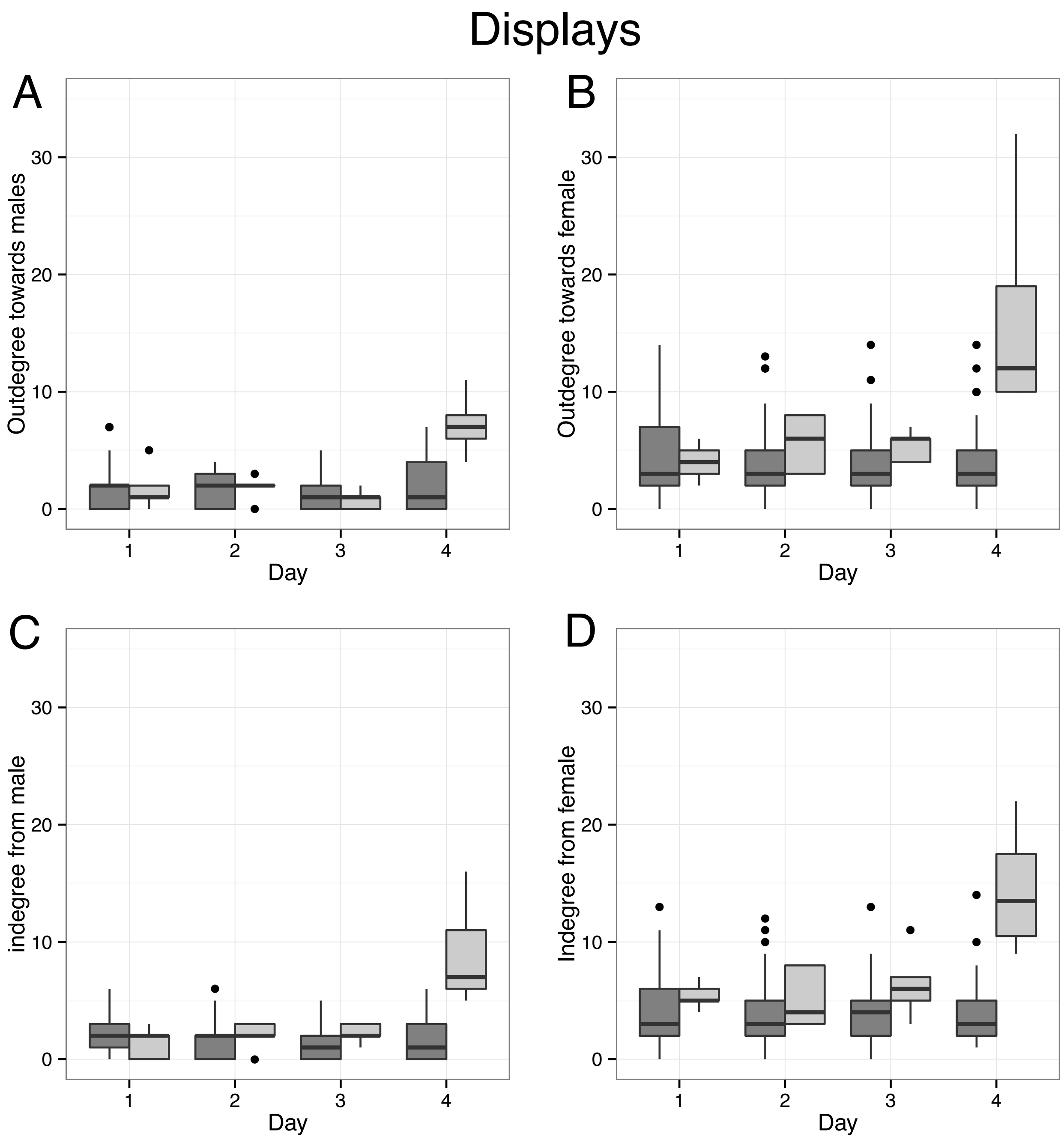

Supplementary Figure S4. Boxplots of display behaviours with manipulated males in light grey and non-manipulated males in dark grey. Horizontal lines represent the median, the box represents the 1st and 3rd quartiles, the whiskers represent the 5th and 95th percentiles. A) Out-degree towards males, B) Out-degree towards females, C) In-degree from males, D) In-degree from females.

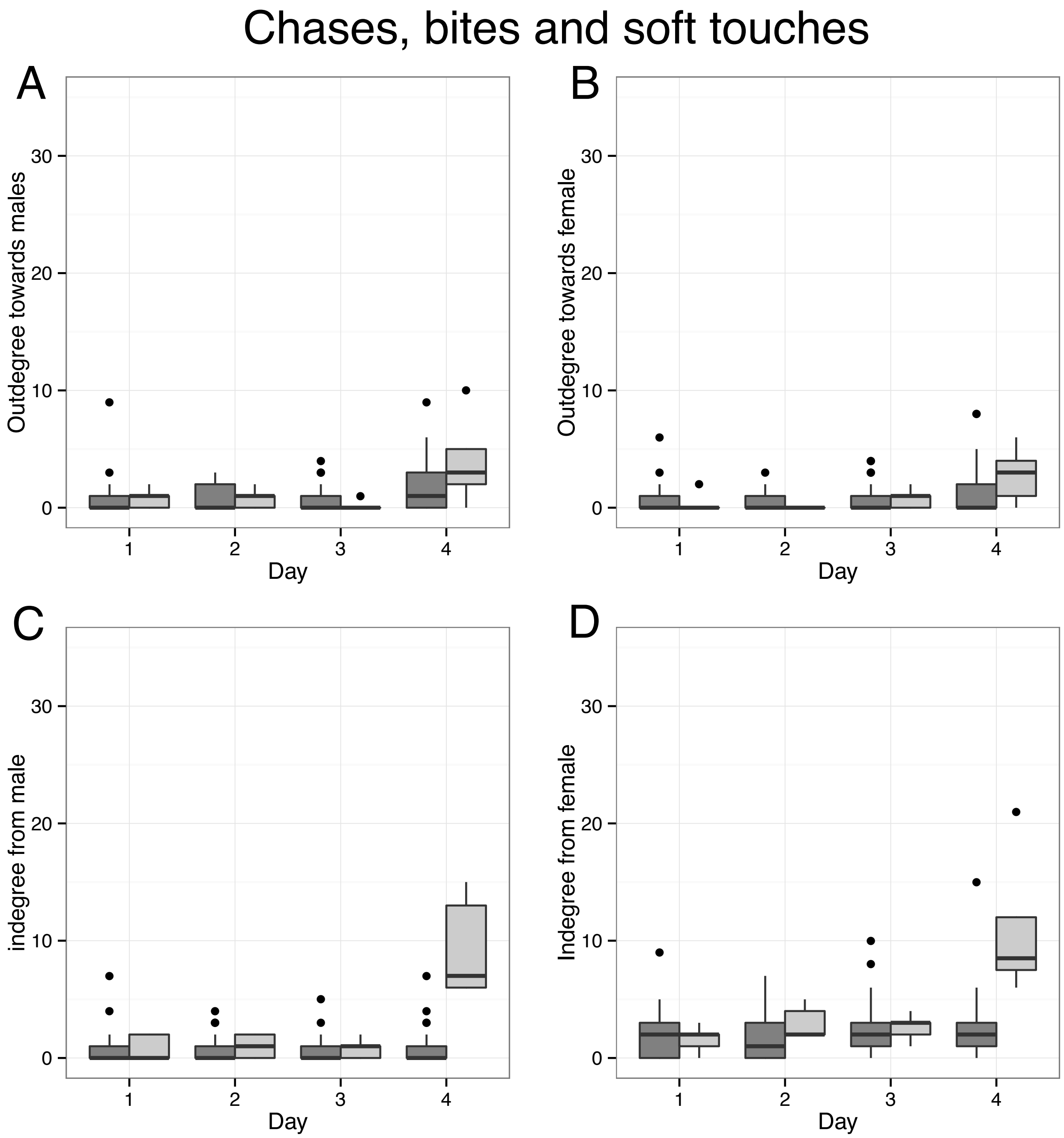

Supplementary Figure S5. Boxplots of direct behaviours (chases, bites and soft touches) with manipulated males in light grey and non-manipulated males in dark grey. Horizontal lines represent the median, the box represents the 1st and 3rd quartiles, the whiskers represent the 5th and 95th percentiles. A) Out-degree towards males, B) Out-degree towards females, C) In-degree from males, D) In-degree from females.

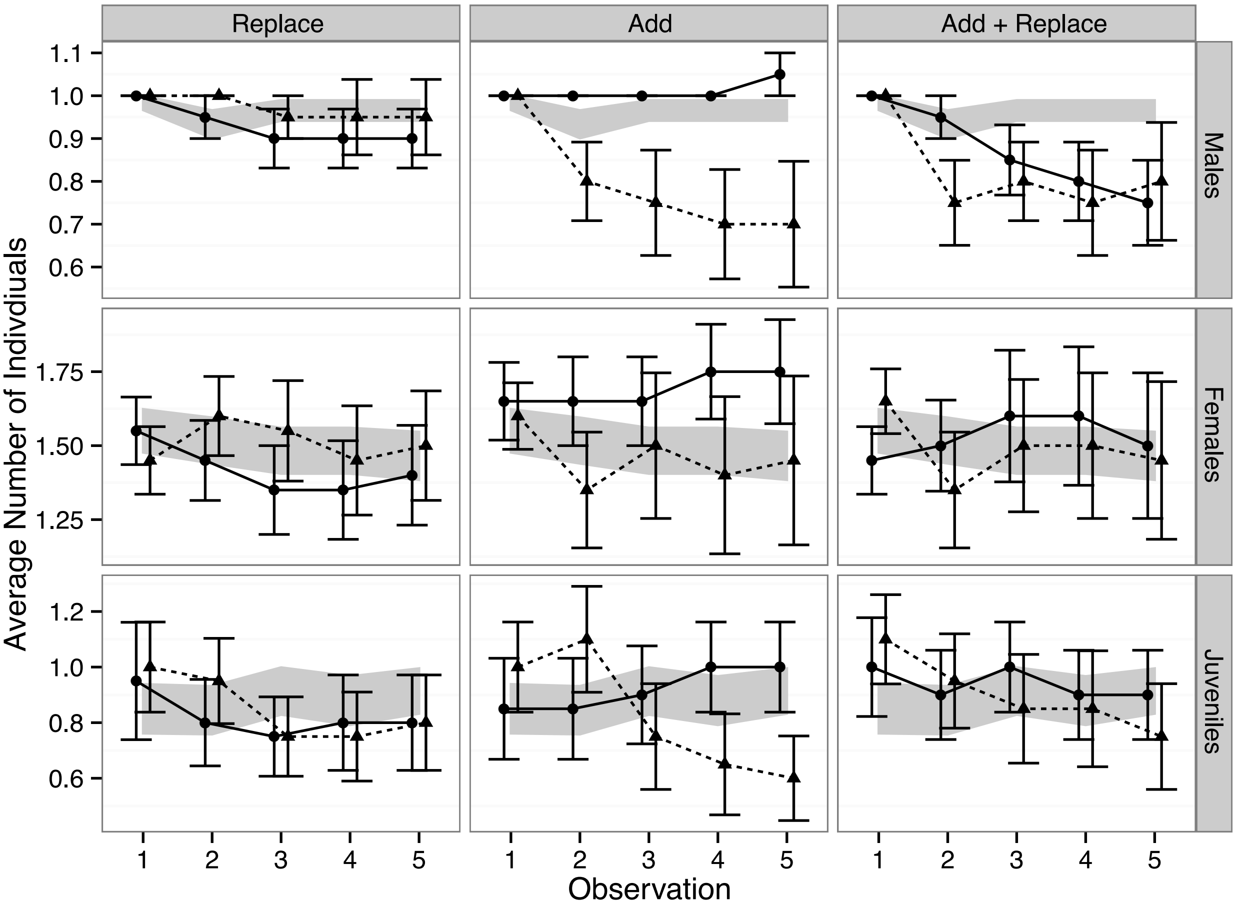

Supplementary Figure S6. The mean (+/- s.e.) number of individuals within each group at each observation (measured every three days) for a total of five observations with all groups included. Data are grouped by treatments which replaced two (solid line) or four (dashed line) existing broken shells with intact shells (***Replace***), added two (solid line) or four (dashed line) intact shells (***Add***), or did both (***Add/Replace***). The grey polygon represents mean (+/- s.e.) for control groups.

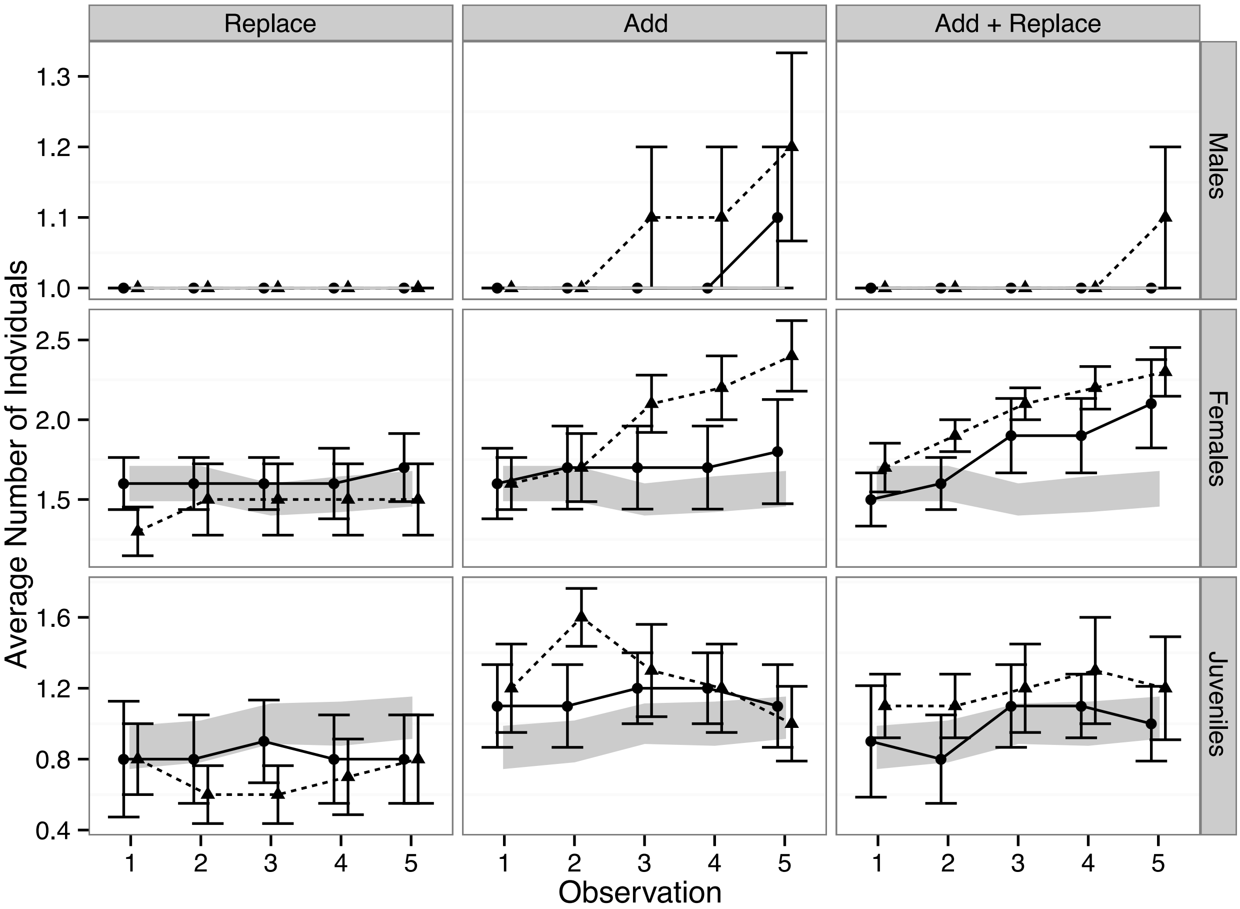

Supplementary Figure S7. The mean (+/- s.e.) number of individuals within each group at each observation (measured every three days) for a total of five observations, omitting groups that were evicted and balancing the comparison such that n = 10 for each group. Data are grouped by treatments which replaced two (solid line) or four (dashed line) existing broken shells with intact shells (***Replace***), added two (solid line) or four (dashed line) intact shells (***Add***), or did both (***Add/Replace***). The grey polygon represents mean (+/- s.e.) for control groups.

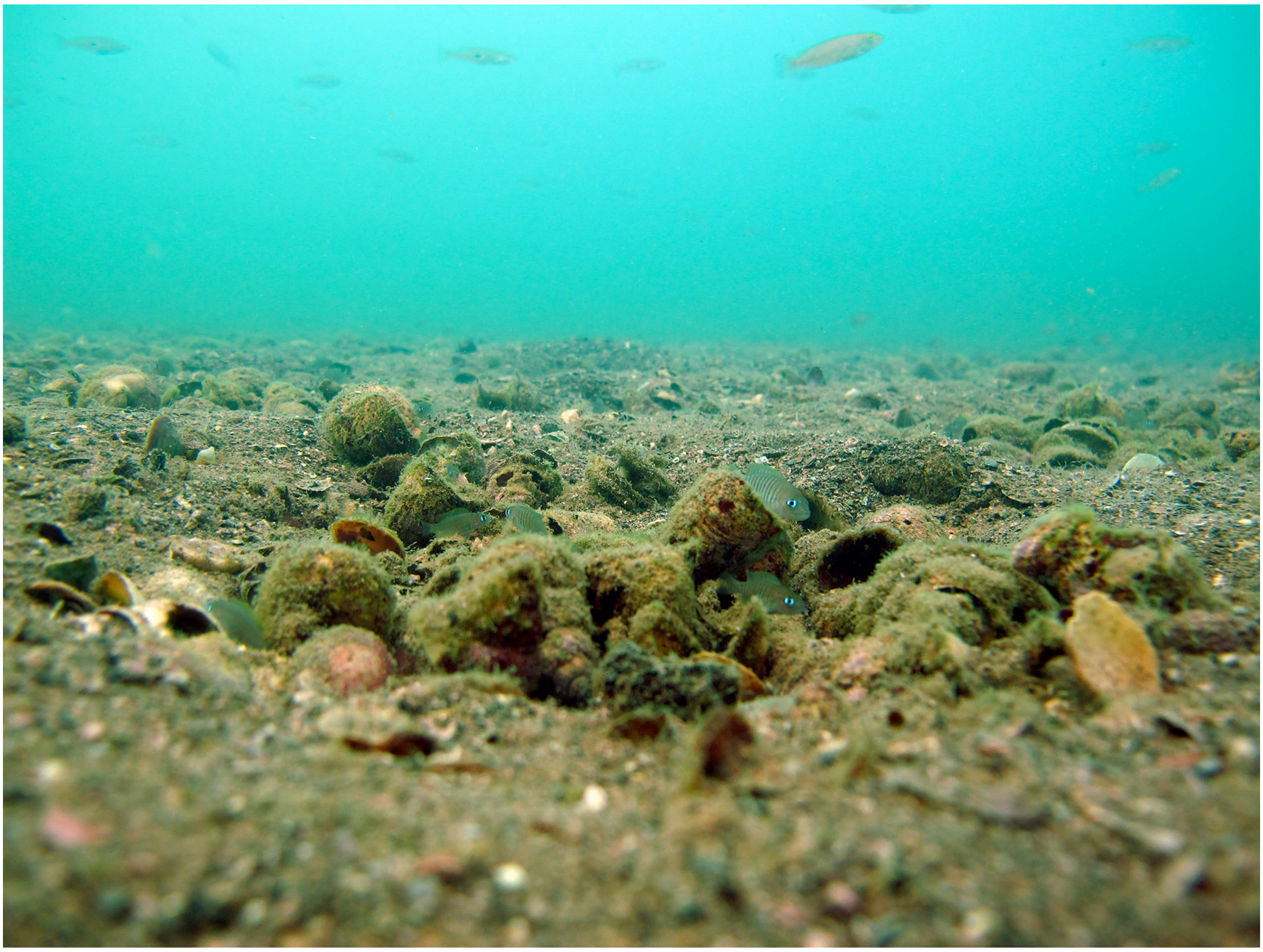

Supplementary Movie S1. Underwater footage of *Neolamprologus multifasciatus* and *Lepidiolamprologus attenuatus* in their natural habitat – *Neothauma* shell beds in Lake Tanganyika. Narrated by Alex Jordan. https://youtu.be/AJuYtrA7tn8

Supplementary Materials References

[1] Kawanabe, H., Hori, M. & Nagoshi, M. 1997 *Fish communities in Lake Tanganyika*. Kyoto, Kyoto University Press.
[2] Kohler, U. 1998 *Zur Struktur und Evolution des Sozialsystems von Neolamprologus multifasciatus (Cichlidae, Pisces), dem kleinsten Schneckenbuntbarsch des Tanganjikasees* Aachen, Shaker Verlag.
[3] Schradin, C. & Lamprecht, J. 2000 Female-biased immigration and male peace-keeping in groups of the shell-dwelling cichlid fish Neolamprologus multifasciatus. *Behavioral Ecology and Sociobiology* **48**, 236-242. (doi:Doi 10.1007/S002650000228).
[4] Schradin, C. & Lamprecht, J. 2002 Causes of female emigration in the group-living cichlid fish Neolamprologus multifasciatus. *Ethology* **108**, 237-248. (doi:Doi 10.1046/J.1439-0310.2002.00775.X).
[5] James, R., Croft, D.P. & Krause, J. 2009 Potential banana skins in animal social network analysis. *Behavioral Ecology and Sociobiology* **63**, 989-997. (doi:10.1007/s00265-009-0742-5).
[6] Pons, P. & Latapy, M. 2005 Walktrap Algorithm. Computing communities in large networks using random walks (long version). In *arXiv:physics/0512106* (
[7] Sopinka, N.M., Fitzpatrick, J.L., Desjardins, J.K., Stiver, K.A., Marsh-Rollo, S.E. & Balshine, S. 2009 Liver size reveals social status in the African cichlid Neolamprologus pulcher. *J Fish Biol* **75**, 1-16. (doi:10.1111/j.1095-8649.2009.02234.x).
[8] Field, A.P. 2013 *Discovering statistics using IBM SPSS Statistics: and sex and drugs and rock 'n' roll*. London, Sage publications.
[9] Holm, S. 1979 A Simple Sequentially Rejective Multiple Test Procedure. *Scand J Stat* **6**, 65-70.
