# Supplementary figures and images for: The social and ecological costs of an ‘over-extended' phenotype
Source: Proc Biol Sci. 2016 Jan 13;283(1822):20152359. doi: 10.1098/rspb.2015.2359 (PMC4721094; doi:10.1098/rspb.2015.2359)

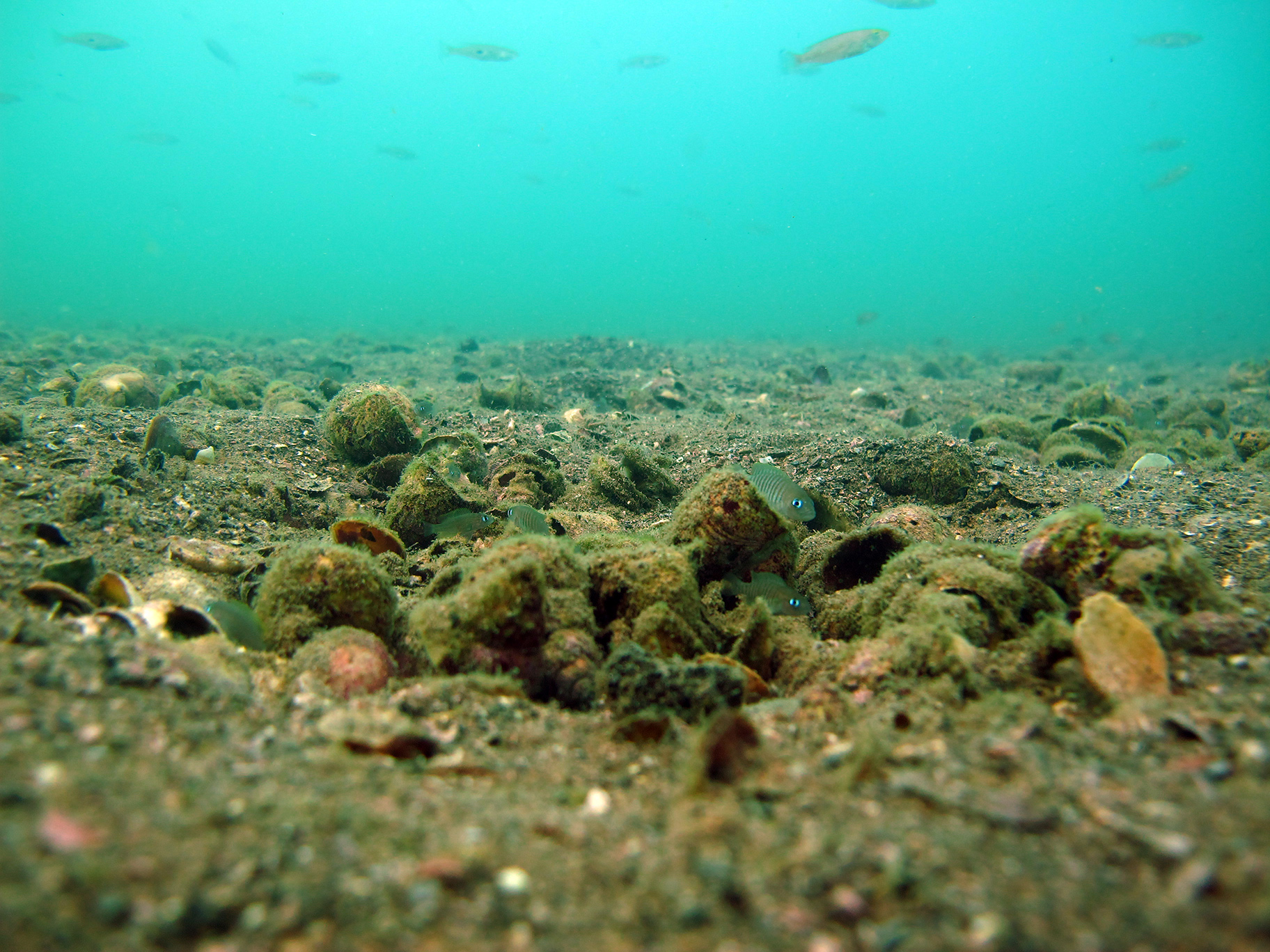

Supplement: Photo of Neolamprologus multifasciatus in Lake Tanganyika [file rspb20152359supp2.jpg]

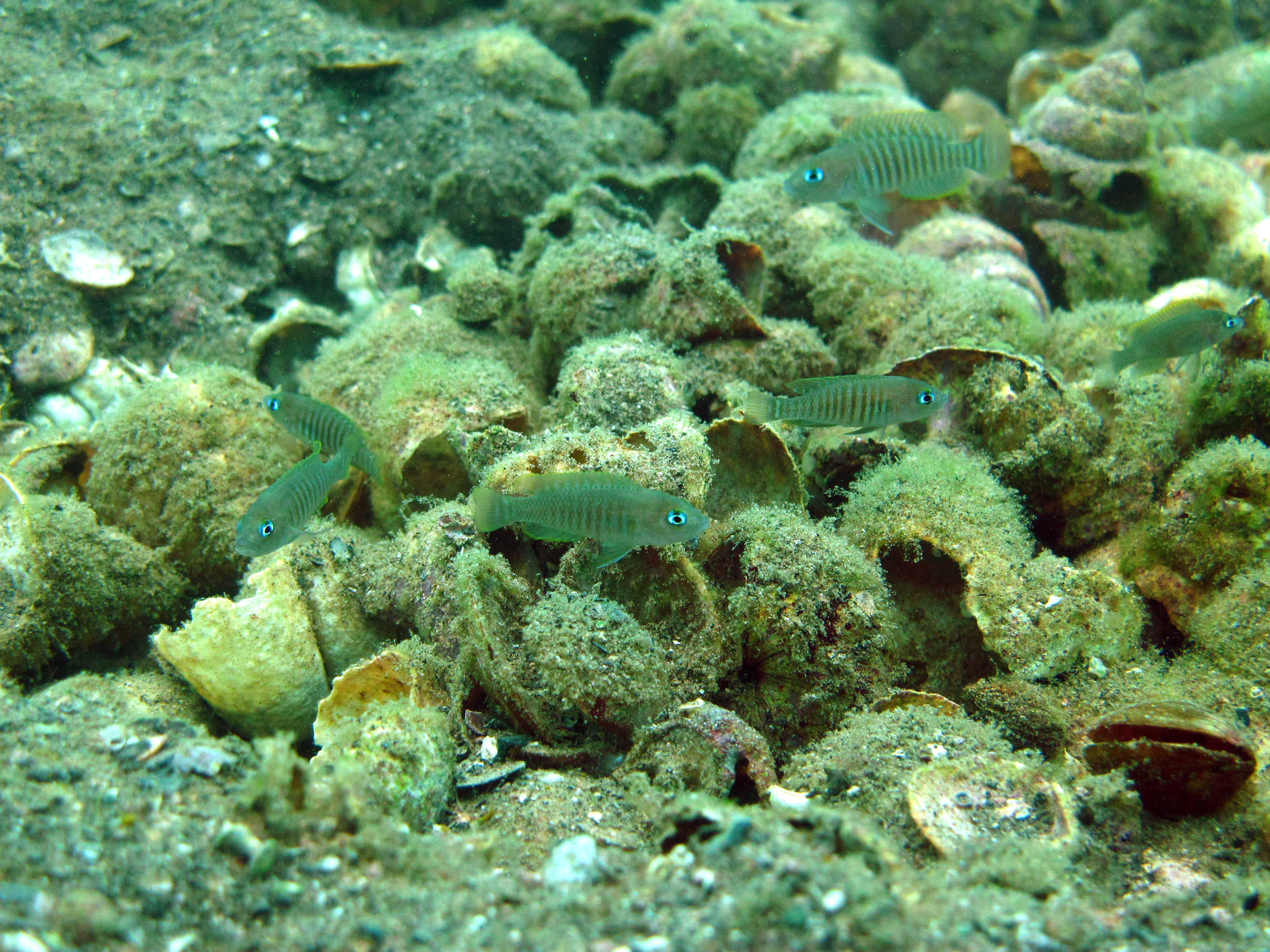

Supplement: Photo of Neolamprologus multifasciatus in Lake Tanganyika [file rspb20152359supp3.jpg]
